# Hospital Cookery

**Published:** 1924-11

**Authors:** 


### Hospital Cookery.

Sir,—Your correspondent, "A La Lanterne," asks what is to be done about the abominable food so often placed before hospital patients. The first step surely is to convince the authorities themselves that variety is an essential in catering not only for patients but for nurses also. In his last report on the Voluntary Hospitals, the late Sir Napier Burnett spoke forcibly of the bad effects of monotonous feeding, yet still we hear of institutions where roast mutton and rice pudding form the menu at least four days out of seven. The idea that hot boiled beef is not very acceptable on a warm summer day or that salads may be a welcome change from the inevitable potato never seems to enter the heads of those responsible for the kitchen department. The two things which middle-class patients find hardest to endure in hospital are the absurd time at which they are awakened and the unattractive and often ill-served food. No pains are spared to make people well again, but it is apparently considered proper that the process should be as uncomfortable as possible in minor ways.

